# β-Eudesmol, an Oxygenized Sesquiterpene, Reduces the Increase in Saliva 3-Methoxy-4-Hydroxyphenylglycol After the “Trier Social Stress Test” in Healthy Humans: A Randomized, Double-Blind, Placebo-Controlled Cross-Over Study

**DOI:** 10.3390/nu11010009

**Published:** 2018-12-20

**Authors:** Kazuaki Ohara, Akane Misaizu, Yuji Kaneko, Takafumi Fukuda, Mika Miyake, Yutaka Miura, Hisayoshi Okamura, Jumpei Yajima, Akira Tsuda

**Affiliations:** 1 Research Laboratories for Health Science and Food Technologies, Kirin Company, Ltd., 1-13-5, Fukuura, Kanazawa-ku, Yokohama 236-0004, Japan; Akane_Misaizu@kirin.co.jp (A.M.); Yuji_1_Kaneko@kirin.co.jp (Y.K.); Takafumi_Fukuda@kirin.co.jp (T.F.); m-miyake@kirin.co.jp (M.M.); yu-miura@kirin.co.jp (Y.M.); 2 Cognitive and Molecular Research Institute of Brain Diseases, Kurume University, 67 Asahi-machi, Kurume, Fukuoka 830-0011, Japan; okamura_hisayoshi@med.kurume-u.ac.jp; 3 Department of Human Studies, Beppu University, Beppu, Oita 874-8501, Japan; yajima@nm.beppu-u.ac.jp; 4 Department of Psychology, Kurume University, 1635 Mii-machi, Kurume, Fukuoka 839-8502, Japan; tsuda_akira@kurume-u.ac.jp

**Keywords:** β-Eudesmol, mental stress, saliva 3-methoxy-4-hydroxyphenylglycol, Trier Social Stress Test (TSST)

## Abstract

Hops, the immature inflorescences of the female hop plant (*Humulus lupulus* L.) are one of the main components of beer and provides flavor and bitterness. β-Eudesmol, an oxygenated sesquiterpene, is reported to accumulate in a particular hop cultivar. Recently, we revealed that β-Eudesmol ingestion affected autonomic nerve activity in an animal model. The effect on humans has not been elucidated, therefore, we investigated the effects of β-Eudesmol on reducing objective and subjective markers related to sympathetic nerve activity after the application of mental stress in healthy participants. Fifty participants (male and female aged 20 to 50 years) were randomly assigned to two groups. Five minutes before taking the Trier Social Stress Test (TSST) as a mental stressor, participants in each group ingested a beverage containing β-Eudesmol, the active beverage, or a placebo beverage that did not contain β-Eudesmol. Saliva 3-methoxy-4-hydroxyphenylglycol (MHPG), a major product of noradrenaline breakdown and a representative marker of sympathetic nerve activity, was significantly lower just after the TSST in the active group compared with the placebo group. Saliva cortisol, a marker of the endocrine stress response system, was not significantly different between the two groups. No adverse events related to test beverage ingestion were observed. This is the first experimental evidence of β-Eudesmol effect for mental stress in human.

## 1. Introduction

Mental stress has been defined as “a set of events in the social milieu which modify steady state conditions so as to activate adaptive mechanisms” [1]. Mental stress itself is not a disorder; however, it affects numerous biological systems, such as autonomic nervous system [2], hypothalamic-pituitary-adrenal (HPA) axis [3], the immune system [4], and increases the risk of mental illnesses such as anxiety, nervousness, symptoms of depression [5], post-traumatic stress disorder [6], and irritable bowel syndrome [7]. The prevalence of mental stress has rapidly increased around the world and intensive research has been conducted into the development of drugs and functional foods which can prevent or reduce mental stress. Natural medications are thought to be mild and safe methods in comparison to conventional medicines. Some natural products have been reported to be effective in reducing mental stress responses in humans [8,9,10,11]. For example, it was reported that L-theanine intake resulted in a reduction in the heart rate responses to an acute stress task, suggesting that it affects autonomic nervous system [8]. However, evidence from clinical trials of natural products is still limited. 

β-Eudesmol, an oxygenated sesquiterpene, has been found in medicinal plants, such as *Atractylodes* [12], *Teucrium* [13], and *Anaxagorea* [14], and accumulates in edible plants, especially a particular hop cultivar [15] and *Eucalyptus* [16]. Some of the physiological effects of β-Eudesmol, determined using a mammal model, have been reported; for example, anti-angiogenic activity, anti-tumor activity, blocking action of succinylcholine on acetylcholine-activated channel activity, and activation of transient receptor potential channels [15,17,18,19], however, effectiveness for humans has not been well studied. It was recently shown that oral administration of β-Eudesmol could affect autonomic nerve activity, resulting in suppression of the adrenal efferent sympathetic nerve system in rats [20]. The effects of β-Eudesmol on autonomic nerve activity response to stressors, and its effectiveness in humans, have also not yet been elucidated in a clinical trial. 

To confirm the effect of β-Eudesmol on mental stress in humans, we performed a placebo-controlled double-blind cross-over study. Healthy adult male and female volunteers ingested water or β-Eudesmol-containing water, just before taking the Trier Social Stress Test (TSST), which is a laboratory-based mental stress test [21]. TSST is utilized as an acute mental stress protocol to study the stress response in human, which was designed in the early nineties. Briefly, participants are introduced a role-playing scenario after a resting period in the laboratory. In the test, a speech (job interview) and a mental arithmetic task (serial subtraction) are performed in front of assessors. Following these tasks, participants rest and post-stressor measurements are carried out. Mental stress related biomarkers are affected due to mental stress caused by TSST [3,22]. In this study, saliva samples before and after stress exposure were analyzed for concentrations of 3-methoxy-4-hydroxyphenylglycol (MHPG) a marker used for sympathetic nerve activity and cortisol, a marker of hypothalamic-pituitary-adrenal (HPA) axis activity. Subjective evaluations were also carried out by questionnaire. In addition, we evaluated the safety of β-eudesmol ingestion in this study.

## 2. Materials and Methods

### 2.1. Study Procedures

The protocol (Protocol No. HR-2016-KR03) was approved by the Institutional Review Boards of the Ethical Committees at the Oriental Ueno Detection Center, General Incorporated Association and Oriental Occupational Health Association, Tokyo Branch (Tokyo, Japan), in accordance with the ethical standards established in the Helsinki Declaration and the ethical guidelines for epidemiological research of the Ministry of Education, Culture, Sports, Science, and Technology, and the Ministry of Health, Labor, and Welfare in Japan. This study was registered with the UMIN Clinical Trials Registry as UMIN000020896 (title: Study of the effects of beverage containing plant ingredient on mental stress), and was conducted in compliance with the protocol. Written informed consent was obtained from all participants. This study was performed by a contract research organization, TES holdings Co., Ltd. (Tokyo, Japan) from January 2016 to June 2016 at the TKP Ichigaya Conference Center (Tokyo, Japan).

Study ID number and website: UMIN000020896, https://upload.umin.ac.jp/cgi-bin/ctr/ctr_view_reg.cgi?recptno=R000024109.

### 2.2. Participants

The entry criteria were as follows: (1) male and female subjects aged between 20 to 50 years old at the time of registration, (2) no history of chronic disease, (3) has feelings of mental stress, such as tension, blushing, increased heartbeat when talking in front of an audience, (4) agreeing to participate voluntarily and in writing, after receiving sufficient explanation of the purpose and content of this study, (5) able to attend the designated examination date and receive examination, and (6) judged able to participate by the study physician. The exclusion criteria were as follows: (1) receiving treatment for a disease, (2) regularly taking drugs or quasi-drugs other than for disease treatment purposes, (3) receiving treatment for, or have a history of, serious disease and/or thyroid gland disease, adrenal gland disease, and/or metabolic disorder, (4) receiving treatment related to psychological stress and/or using related pharmaceutical products, supplements and health foods, (5) taking antihistamine drugs; xerostomia, or have subjective symptoms of xerostomia, (6) receiving treatment for, or have a history of, drug addiction and/or alcoholism, (7) systolic and diastolic blood pressures over 160 mmHg and 100 mmHg, (8) have a drug allergy, food allergy, and/or possibility of allergy symptom onset, (9) have subjective symptoms of anemia, (10) predicted to, or have, life events which may affect the participant’s mental condition, (11) involved in a profession which includes public speaking or a sales position, (12) smoker, (13) unable to stop drinking alcohol during the day before, and at the end of each test on each inspection day, (14) unable to stop the intake of caffeine-containing foods or beverages from morning until the end of the test on each inspection day, (15) unable to sleep at night due to shift work or other reasons, (16) individuals whose work involves taking a holiday during the week, (17) individuals who do not naturally wake between 6:00 and 9:00 a.m., (18) individuals who and whose family work for a company manufacturing or selling healthy foods or functional foods, (19) individuals planning to participate in other clinical studies during this study period, (20) females planning a pregnancy during the current study or are pregnant or lactating, (21) individuals who have a significantly deviated body measurement value, (22) individuals considered unsuitable from their background investigation, and (23) participants judged as unsuitable by the principal investigator. Fifty subjects finally participated in this study.

### 2.3. Test Beverages

Beverages of 190 mL were prepared, containing either 950 ng of β-Eudesmol (active beverage), or no β-Eudesmol (placebo beverage). β-Eudesmol was provided by Takasago International Corporation (Tokyo, Japan). The nutritional compositions of the test beverages are shown in Table 1. The study controller confirmed that there were no discernible differences between the appearance and taste of the two test beverages before the allocation.

### 2.4. Study Design

The TSST was conducted according to previous reports [21]. A randomized double-blind, placebo-controlled cross-over study was conducted over five weeks, consisting of a screening day, a first beverage ingestion day, a two-week washout period, and a second beverage ingestion day. The screening test for study eligibility was performed over 16 days before the beginning of the first beverage ingestion period and eligible participants visited the TKP Ichigaya Conference Center for all of the tests. Measurements of the anthropometric and circulatory parameters were only conducted at the screening test. Interviews by a physician were conducted at every test. To minimize the circadian variations of biological markers, all experimental sessions started after 11:00 and ended before 17:30. Subjects arrived at least 30 min before the start of the study and this was named the habituation period. Subjects ate a standardized meal as lunch during the habituation period.

The study controller randomly assigned participants in a 1:1 ratio into two groups with random numbers, and stored the assignment list in a locked container. All participants, investigators, and study personnel, except for the controller, were blinded to the assignment list throughout the study. Participants consumed one test beverage just before the TSST on ingestion day. Participants were instructed to continue their usual eating, exercise, sleeping, smoking and drinking habits throughout the study. Use of oral medication, dietary supplements and functional foods affecting mental stress were prohibited. Job interviews and public speaking were prohibited during the test period. On the day before the test, participants were prohibited from drinking alcohol and were asked to finish their evening meal 12 h before the test commenced, after which eating (except for the prepared standardized meal) and drinking (except water) were prohibited until the test was complete. Smoking was also prohibited on the test day until the test was complete. 

Discontinuance criteria for the study participants were as follows: (1) injury to the participant, (2) difficulty in continuing the study due to a serious adverse event or accident, (3) continuous or serious noncompliance of the protocol, (4) pregnancy, or (5) anything which the site investigator judged necessary to cause discontinuance of the study.

### 2.5. Measurements of Anthropometric and Circulatory Parameters

Height, body weight, body fat ratio (dc-320 Tanita, Tokyo, Japan), systolic blood pressure, diastolic blood pressure and pulse rate (H55 TERUMO CORPORATION, Tokyo, Japan) were measured at the screening test. BMI was calculated from height and body weight.

### 2.6. Daily Life Diary

The participants recorded subjective symptoms and daily activities, including: wake time, bed time, any actions taken to relieve stress or fatigue and drug intake, in a diary every day during the test period. To avoid any influence of the menstrual cycle on the salivary biological markers, each participant was checked for the phase of their menstrual cycle.

### 2.7. Interview

Participants were interviewed about their physical condition and subjective symptoms by the study physician before and after each test.

### 2.8. Endpoints

The primary endpoints were improvement in salivary MHPG and cortisol levels after TSST, and secondary endpoints were improvements in salivary chromogranin A [23], State-Trait Anxiety Inventory-Form JYZ [24], and Dundee Stress State Questionnaire III [25]. Chromogranin A is an acidic glycoprotein released with catecholamine from the adrenal medulla and the sympathetic nerve endings and has been used as a stress marker in saliva. Safety endpoints were any occurrence of an adverse event. Evaluation of safety was conducted by doctor’s inquiries to participants in test days, and diary during the examination period written by participants. When an adverse event occurred, the site investigators conducted a follow-up survey until the event disappeared or recovery began (last date of the follow-up survey: 20 March 2016).

### 2.9. Measurements of Salivary Parameters

Saliva was collected using a swab and centrifuge tube (Salimetrics Oral Swab (SOS), SALIMETRICS, Carlsbad, CA, USA) at Ichigaya Conference Center. And the resulting saliva was divided and kept frozen at −80 °C until use at Kirin Company and Kurume University, respectively. Saliva MHPG was measured by gas chromatography coupled with mass spectrometry (Hitachi–M80B, Hitachi, Japan) as described previously at Kurume University [26]. Standard solutions containing 1, 5, and 10 ng of free–MHPG in 10 mL of Ringer’s solution were prepared, and used to derive the calibration curve. GC-MS analysis was carried out using a Hitachi M–80B (Hitachi, Tokyo, Japan) double focused mass spectrometer interfaced to a data acquisition system. Helium was used as a GC carrier gas (40 mL/min). The GCMS interface oven and transfer line were set at 260 °C. A megabore column coated with DB-1 (0.53 mm i.d. × 15 m, J and W, Co., Santa Clara, CA) was used. The oven temperature was increased at a rate of 25 °C/min from 125 °C to 220 °C. The electron emission current was 100 mA and the electron energy was 70 eV. For the electron ion monitoring study, m/z 472 and m/z 475 were monitored. The ratio of the peak areas was used for estimation of the amount of MHPG from the calibration curve. The data are expressed as ng/mL of MHPG. Saliva cortisol and chromogranin A were measured by commercially available kits according to the manufacturer’s instructions (Salivary cortisol EIA kit, (SALIMETRICS) and Human Chromogranin A EIA, (Yanaihara Institute Inc., Shizuoka, Japan), respectively at Kirin Company (Tokyo, Japan).

### 2.10. Statistical Analysis

Data were expressed as the mean plus SEM. Dunnett’s test was used to evaluate the change from the data at −5 min. Paired Student′s *t*-test was used to compare the difference between the two groups at each time point. Wilcoxon signed-rank test was used for all subjective assessments. Differences were considered significant at *p* < 0.05.

## 3. Results

### 3.1. Study Design and Background of the Participants

**Figure 1:**
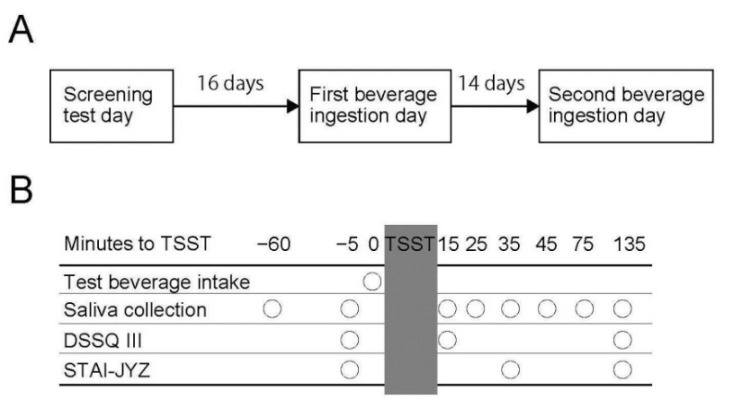
Schematic representation of test schedule. (**A**) Whole test schedule of this study. (**B**) Schedule of the test day. Timing of test beverage ingestion, saliva collection and questionnaire are shown by circles. DSSQIII, Dundee Stress State Questionnaire III; STAI-JYZ, State-Trait Anxiety Inventory-Form JYZ; TSST, Trier Social Stress Test.

**Figure 2:**
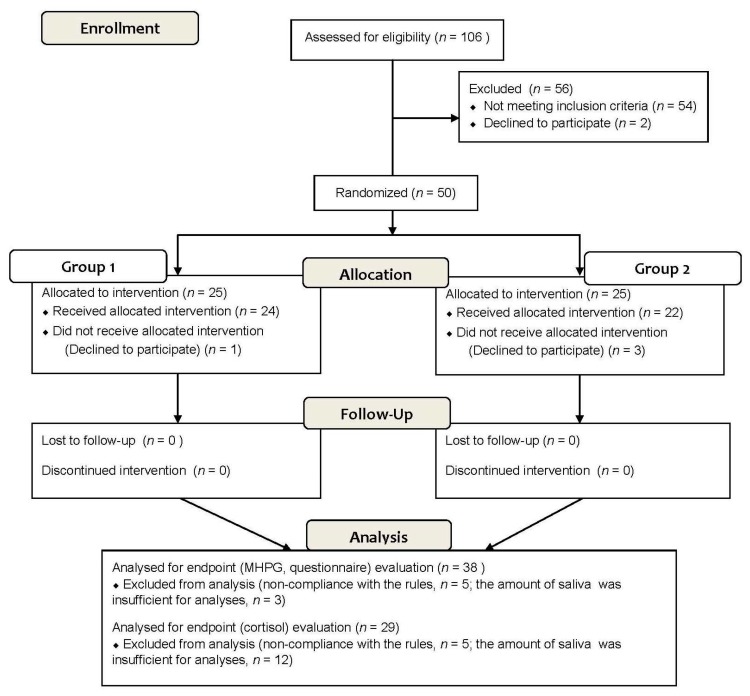
Flow diagram of the progress through the study.

The study design is presented in Figure 1, and a detailed explanation is given in the materials and methods section. The time schedule of the test day is shown in Figure 1B; the TSST start time is defined as 0 min. The flow of participants enrolled in this study is shown in Figure 2. Out of 106 participants which underwent the screening test, 56 participants were excluded so that 50 participants (33 male and 17 female) were randomized into two groups (25 participants in each group). Four participants dropped out of the study due to personal problems not related to the study. Among the 46 remaining participants who finished the study, eight participants were excluded by the site investigators on the basis of their primary and secondary endpoint evaluations. Exclusion was performed before the release of the allocation list and was in accordance with the exclusion criteria determined before the study. Exclusion was due to: failure to obey compliance rules (five participants) and insufficient amount of saliva for MHPG analyses (three participants). The baseline characteristics of the 38 participants are shown in Table 2, indicating that age met entry criteria, and blood pressure was lower than exclusion criteria. Statistical analyses of primary endpoint, MHPG evaluation, and secondary endpoints (State-Trait Anxiety Inventory-Form JYZ, and Dundee Stress State Questionnaire III) were conducted on 38 participants (25 male and 13 female). Among the 38 participants, nine participants were excluded due to in sufficient amount of saliva for analysis on the basis of their saliva cortisol evaluation. Of the remaining 29 participants, 16 were also excluded due to insufficient amount of saliva for chromogranin A evaluation. Consequently, statistical analyses of saliva cortisol and chromogranin A were conducted on 29 and 13 participants, respectively.

### 3.2. Primary Endpoint

**Figure 3:**
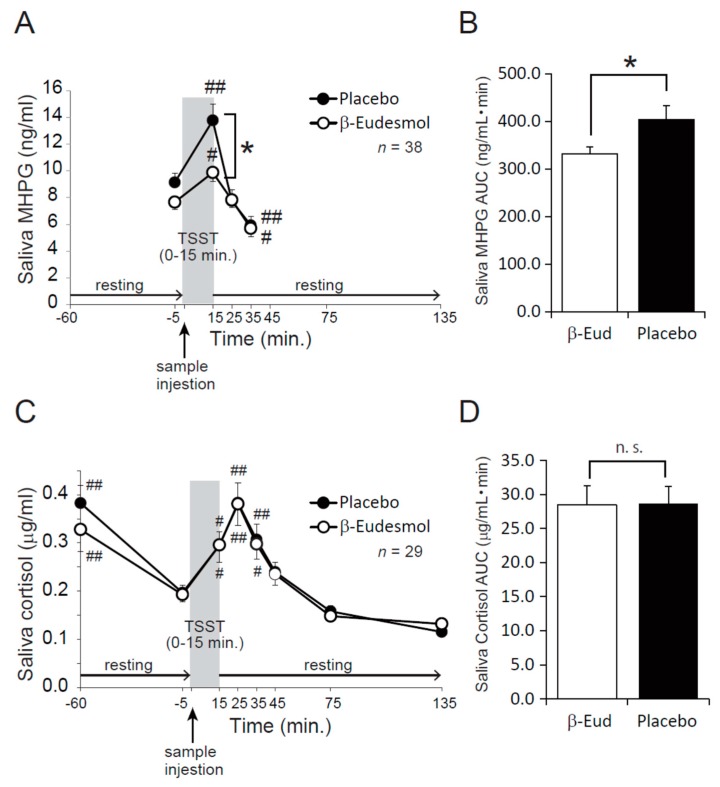
Effects of β-Eudesmol on saliva 3-methoxy-4-hydroxyphenylglycol (MHPG) and cortisol. (**A**) Effects of β-Eudesmol on saliva MHPG. Saliva MHPG levels were measured at −5, 15, 25, and 35 min. (**B**) Area under the curve (AUC) of saliva MHPG. (**C**) Effects of β-eudesmol on saliva cortisol. Saliva cortisol levels were measured at −60, −5 15, 25, 35, 45, 75, and 135 min. (**D**) AUC of saliva cortisol. Data are expressed as means ± SEM. β-Eud, β-Eudesmol; n.s., not significant; TSST, Trier Social Stress Test *, *p* < 0.05 between two groups. #, *p* < 0.05 in comparison to -5 min. ##, *p* < 0.01 in comparison to −5 min.

Figure 3A shows the change in concentration of saliva MHPG levels during the tests. Saliva MHPG was significantly increased in both groups just after taking the TSST when compared with before the test started (−5 min). Saliva MHPG levels were reduced in both groups during the 10 min resting period (25 min in Figure 3A), and no significant differences were observed between the groups. Saliva MHPG levels were significantly decreased between the −5 min and 20 min resting time points in both groups (35 min in Figure 3A). Saliva MHPG was significantly lower in the β-Eudesmol group compared with the placebo group just after the TSST; however, levels were significantly increased at the 15 min time point when compared to −5 min in both groups (Figure 3A, *p* < 0.05). No significant differences in saliva MHPG levels were observed between the two groups at the other time points. Figure 3B shows the area under the curve (AUC) for saliva MHPG which was significantly lower in the β-Eudesmol group compared with the placebo group (*p* < 0.05).

Figure 3C shows the change in saliva cortisol concentration during the test. No significant difference was observed between the β-eudesmol group and placebo group during all time points tested. Saliva cortisol was significantly increased in both groups just after the TSST, and at 10 and 20 min rest (35 min and 45 min in Figure 3C) when compared with −5 min. The AUC for saliva cortisol (Figure 3D) shows there was no significant difference between the β-Eudesmol group and placebo group.

### 3.3. Secondary Endpoints

The change in saliva chromogranin A levels throughout the study was shown in Table S1. No significant difference was observed between the β-eudesmol group and placebo group during all time points tested. Saliva chromogranin A was significantly increased in the placebo group at 45 and 135 min after the TSST when compared with −5 min; however, there were no significant differences between −5 min and the measured time points in the β-eudesmol group.

The State-Trait Anxiety Inventory-Form JYZ, and Dundee Stress State Questionnaire III were used to evaluate anxiety and stress state, respectively (Table 3). There were no significant differences in the −5 min values of any parameter in the two questionnaires between the two groups. As shown in Table 3, the TSST significantly increased the scores for state anxiety in both groups; however, no significant differences were observed between the β-Eudesmol group and placebo group. Trait anxiety scores were not significantly affected by the TSST in either group. The Dundee Stress State Questionnaire III showed that the TSST significantly increased “Unpleasant stress” and “Anxiety” scores in both groups; however, no significant differences were observed between the groups. A significant difference in the “Concentration on tasks” score was observed between the placebo group and β-Eudesmol group at 135 min. the β-Eudesmol group showed a significant difference in “Concentration on tasks” score between −5 min and 135 min.

### 3.4. Safety Endpoints

Safety endpoint evaluation was conducted on 46 participants who ingested test beverages at least once. No clinically problematic findings were noted from urinalysis throughout the study. During the study period, 15 adverse events involving 11 participants were reported. The most common adverse event was pollen allergy-like symptoms or headache. Adverse events reported during the study period were as follows: three cases of pollen allergy, three cases of headache, two cases of stomach ache, two cases of muscle ache, two cases of menstrual pain, and one case each of shoulder discomfort, dental pain, and dry eye. All cases were judged to be mild and to have no relation to the test beverages by the site investigators.

## 4. Discussion

We investigated the effect of β-Eudesmol ingestion prior to exposure of mental stress via the TSST model in healthy adult participants. Saliva MHPG and cortisol were significantly increased by the TSST in both groups. Saliva MHPG increased faster than cortisol which may reflect the difference between the two parameters: saliva MHPG indicates autonomic nerve activity [27], and saliva cortisol indicates endocrine system activities. The response pattern against a mental stressor was consistent with previous reports [28,29]. In addition, scores from the State-Trait Anxiety Inventory-Form JYZ showed that the TSST significantly increased anxiety in both groups. These results suggest that mental stress was successfully loaded onto participants by our experiments.

MHPG reflects central nervous system activity, and is relevant to symptoms of anxiety or depression [30]. β-Eudesmol significantly lowered saliva MHPG concentrations after the TSST in adult participants when compared with the placebo group. The level of saliva MHPG is reported to highly correlate with plasma MHPG levels and is therefore an established marker of central noradrenergic metabolism. It is suggested that β-Eudesmol could suppress sympathetic nerve activity which responds to acute mental stress. However, no significant difference in saliva cortisol level was observed between the β-Eudesmol and placebo groups although saliva cortisol was increased in both groups after the TSST. Saliva cortisol was higher at −60 min when compared with the TSST starting time (0 min) in both groups. Saliva cortisol is reported to be affected by dietary components [31], and the higher cortisol levels at −60 min may be due to the standardized meal during the habituation period. These results indicate that β-Eudesmol mainly affects autonomic nerve activities and not HPA-axis activities. Participants were instructed to maintain their normal lifestyle i.e., eating, exercise, sleeping, smoking, and drinking habits, during the study period. Saliva MHPG and cortisol concentrations did not differ between the two groups before the start of test beverage intake, indicating that ingestion of β-Eudesmol reduces saliva MHPG secretion after mental stress induced by the TSST in healthy participants. A significant difference was observed in the MHPG response after the TSST between the groups; however, no significant difference in the subjective evaluations was found between the groups, except the “Concentration on task” from the Dundee Stress State Questionnaire III after 135 min of β-Eudesmol intake. The significant decrease in “Concentration on task” score in the β-Eudesmol group suggests a relaxation effect on subjective feeling, possibly as a result of a reduction in sympathetic nerve activity by β-Eudesmol. Further research is required to reveal any relationship between the “Concentration on tasks” score lowering effect and the physiological effects of β-Eudesmol.

Our previous study revealed that oral-administration of β-Eudesmol affects autonomic nerve activity in an animal model [20], which supports the mechanism involving β-Eudesmol intake lowering the saliva MHPG concentration after mental stress in this study. In an animal model, adrenal efferent sympathetic nerve activity is suppressed after orally administered β-Eudesmol; however, it is not suppressed by subcutaneous administration [20]. It is reported that the scent threshold of β-eudesmol is 10,000 ppb [32]: the active beverage contained 5 ppb β-Eudesmol in this study and could not be distinguished from the placebo beverage by the controller. These results suggest that the organ in which β-Eudesmol first acts may be the digestive tract and reflects autonomic nerve activity. In previous report, β-Eudesmol could stimulate transient receptor potential ankyrin 1 (TRPA1) [15]. The TRPA1 is a calcium-permeable non-selective cation channel which plays a crucial role in the susceptibility to various stimuli [33]. TRPA1 is mainly expressed in sensory nerves and plays an important role in the susceptibility to various stimuli and is also expressed in the digestive tract [34,35]. Reduction of sympathetic nerve activity by oral-administered β-Eudesmol was eliminated in TRPA1 knockout rat [20], suggesting that TRPA1 activation by β-Eudesmol may be involved for reduction of MHPG in this human study. In previous study, l-theanine intake reduced the heart rate responses to an acute stress task, suggesting that l-theanine affects autonomic nerve activity against mental stress. Suggesting mechanism of its effect is to increase γ-aminobutyric acid (GABA) [8]. Whereas the relation of l-theanine and TRPA1 was not reported, it is a future work whether β-eudesmol shows the same effect as the l-theanine, such as reduction of heart rate or increase of GABA.

The TSST has been widely used in the basic understanding of psychological endocrinology [3]. In addition, the TSST has been used to assess the effects of both therapeutic and nonmedical drugs [36], and has also been used to verify the effect of foods and beverages which may relieve mental stress [8,9,10]. Previously, food ingredients with stress response effects have been elucidated using the TSST and measuring saliva and serum cortisol, or adrenocorticotropic hormone (ACTH) elevation as stress markers [10,37]. Our study showed that the TSST increased saliva MHPG as well as saliva cortisol, indicating that the TSST is also able to detect activation of the sympathetic nervous system due to mental stress. Since saliva can be collected noninvasively, saliva MHPG is thought to be a useful marker in developing functional foods or beverages to mitigate acute physiological responses against a stressor. Beverages containing β-Eudesmol may suppress the activation of the sympathetic nervous system and it can be taken by the simple method of a single oral intake just before mental stress. Especially, it may be effective against mental stress caused by socially evaluated environment, since the TSST is a well-established method that can induce social-evaluative threat in the laboratory [38].

All adverse events reported during the study period were judged to be mild and to be unrelated to the test beverages by the site investigators. Thus, it may be assumed that single ingestion of β-Eudesmol at the dose used in this study is safe. In addition, the effects of β-Eudesmol on reducing saliva MHPG was mitigated at the end of the test, indicating that the effect of a single dose of β-Eudesmol is transient and does not persistently affect autonomic nerve activity.

## 5. Conclusions

In conclusion, our study revealed that single ingestion of β-Eudesmol reduces the acute mental stress response, in particular, saliva HMPG secretion indicating sympathetic nerve activity in healthy humans. Intake of β-Eudesmol (950 ng) was sufficient to achieve this effect. Therefore, β-Eudesmol could be a useful and safe tool to prevent mental stress responses and related disorders. The effectiveness of β-Eudesmol on the acute mental stress response was confirmed in this study; the effects on chronic mental stress will be investigated in a future study.

## Figures and Tables

**Table 1:** Nutritional compositions of test beverages (per 190 mL).

	Active Beverage	Placebo Beverage
Energy (kcal)	0	0
Protein (g)	0	0
Lipid (g)	0	0
Available carbohydrate (g)	0	0
Na (mg)	0	0
β-Eudesmol (ng)	950	0

**Table 2:** Baseline characteristics of the participants for evaluation of primary endpoint.

Parameter	Participants
*n*	38
Age (years)	32.2 (1.4)
Height (cm)	167.3 (1.3)
Body weight (kg)	62.9 (1.7)
BMI (kg/m^2^)	22.4 (0.6)
Systolic blood pressure (mmHg)	110.4 (2.1)
Diastolic blood pressure (mmHg)	65.6 (1.6)
Heart rate (bpm)	72.8 (2.0)

Data are expressed as means (Standard error of mean).

**Table 3:** Results of the questionnaires (*n* = 38).

**Dundee Stress State Questionnaire III**					
	Sample	−5 min	15 min	35 min	135 min
Concentration on task	Active Placebo	19.6 (0.7) 18.8 (0.6)	18.6 (0.7) 19.5 (0.7)	- -	17.6 (0.6) **^,#^ 18.7 (0.7)
Unpleasant stress	Active Placebo	17.0 (0.7) 17.1 (0.8)	19.7 (0.8) ** 20.4 (0.8) **	- -	18.3 (0.9) 17.5 (0.9)
Anxiety	Active Placebo	12.4 (0.7) 12.4 (0.7)	13.6 (0.7) * 13.7 (0.5) *	- -	13.6 (0.6) 13.0 (0.6)
**State-Trait Anxiety Inventory-Form JYZ**					
	Sample	−5 min	15 min	35 min	135 min
State Anxiety	Active Placebo	47.0 (1.2) 46.7 (1.3)	- -	52.4 (1.5) ** 49.9 (1.9) *	39.2 (1.3) ** 38.9 (1.3) **
Trait Anxiety	Active Placebo	47.5 (1.4) 47.7 (1.5)	- -	48.7 (1.4) 47.9 (1.7)	47.2 (1.5) 47.0 (1.6)

Data are expressed as means (SEM). #, *p* < 0.05 between two groups. *, *p* < 0.05 in comparison to −5 min. **, *p* < 0.01 in comparison to −5 min.

## Supplementary Materials

The following are available online at http://www.mdpi.com/2072-6643/11/1/9/s1, Table S1: Saliva chromogranin A concentration during the TSST (*n* = 19).

## Abbreviations

MHPG	3-Methoxy-4-hydroxyphenylglycol
TRPA1	Transient receptor potential ankyrin 1
TSST	Trier Social Stress Test
