# Supplementary material for: β-Eudesmol, an Oxygenized Sesquiterpene, Reduces the Increase in Saliva 3-Methoxy-4-Hydroxyphenylglycol After the “Trier Social Stress Test” in Healthy Humans: A Randomized, Double-Blind, Placebo-Controlled Cross-Over Study
Source: Nutrients. 2018 Dec 20;11(1):9. doi: 10.3390/nu11010009 (PMC6356403; doi:10.3390/nu11010009)
Supplement: Supplementary file 1 [file nutrients-11-00009-s001.docx]

**Supplemental Table . Saliva chromogranin A concentration during the TSST (*n* = 19)**

| Time | Active beverage  pmol/mg protein | Placebo beverage  pmol/mg protein |
| --- | --- | --- |
| -60 min | 8.00 (3.91) | 2.32 (0.66) |
| -5 min | 5.89 (1.99) | 4.47 (1.48) |
| -Test beverage intake-  -TSST- | | |
| 15 min | 21.3 (14.6) | 6.05 (1.69) |
| 25 min | 8.32 (4.53) | 7.91 (4.08) |
| 35 min | 5.45 (1.84) | 3.83 (0.99) |
| 45 min | 9.72 (3.76) | 10.5 (3.73)^#^ |
| 75 min | 10.3 (4.85) | 6.48 (2.22) |
| 135 min | 11.6 (5.64) | 7.64 (3.17)^#^ |

^#,^ p<0.05. Dunnett's test was used to evaluate the change from -5 min.
